# Successful use of picosecond laser treatment for seborrheic keratosis in three Asian patients

**DOI:** 10.1016/j.jdcr.2024.04.006

**Published:** 2024-04-16

**Authors:** Madisen A. Swallow, May Elgash, Sa Rang Kim, Kathleen C. Suozzi

**Affiliations:** a Yale School of Medicine, New Haven, Connecticut; b Department of Dermatology, Yale School of Medicine, New Haven, Connecticut

**Keywords:** laser therapy, picosecond laser, seborrheic keratosis, skin of color

## Introduction

Seborrheic keratosis is the most common benign tumor of the epidermis and is clinically defined by their sharp demarcation from the neighboring skin, resulting in its distinctive “stuck-on” appearance.^1^ Although benign, many patients desire to have these lesions removed due to site irritation, pruritus, or cosmesis.^2^ Common treatment options for seborrheic keratosis include cryotherapy, curettage, blunt dissection, and the use of laser therapy. Treatment with erbium-doped yttrium-aluminum-garnet laser has demonstrated a significantly lower rate of post treatment hyperpigmentation when compared to cryotherapy.^3^

Historically, there has been a hesitancy to use laser treatments for patients with skin of color due to an increased risk of postinflammatory dyspigmentation or scarring.^4^^,^^5^ In this series, we demonstrate successful use of 532 nm picosecond laser (PicoWay Candela) in the treatment of seborrheic keratosis in three Asian patients. This laser delivers ultrashort pulse durations in the 250 to 450 picosecond range.

## Case reports

Three Asian patients were treated for seborrheic keratoses on the face with a 532-nm picosecond laser. Lesions were diagnosed as seborrheic keratoses based on clinical exam, and a thorough history was obtained prior to treatment to exclude other forms of pigmentation such as melasma or post-inflammatory hyperpigmentation. When there was diagnostic uncertainty regarding the diagnosis (lentigo vs macular seborrheic keratosis), a test spot was performed. The exact test spot locations varied by patient; however, all were performed away from the central areas of the face. Patients were re-evaluated six-eight weeks later at which time treatment was carried out if no adverse effects were noted. All test spots were completed without any postinflammatory hypopigmentation or hyperpigmentation.

**Fig 1:**
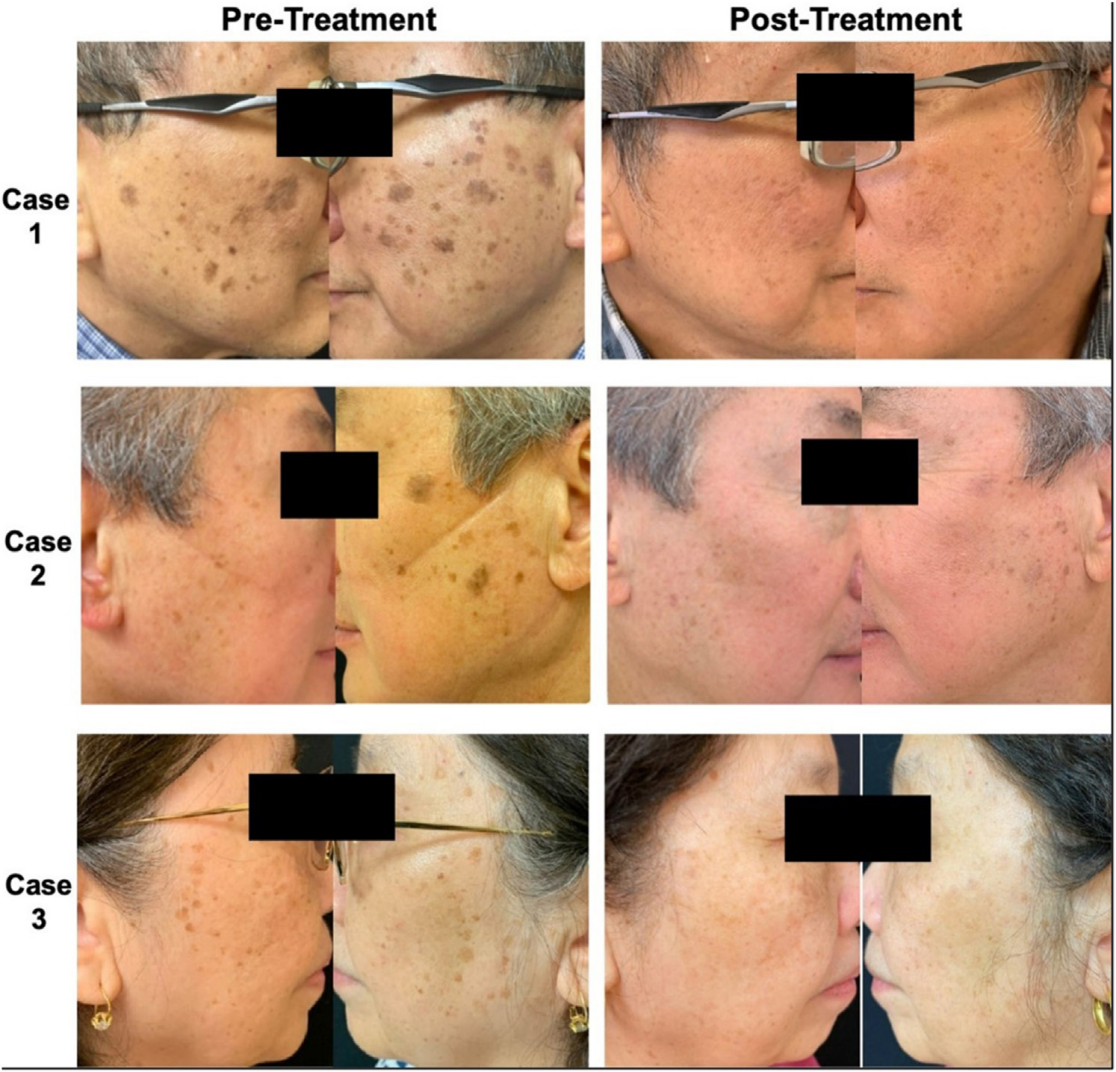
Clinical improvement of three Asian patients with 532 nm picosecond laser treatment.

No topical anesthesia was used as treatment was well-tolerated. The treatment endpoint was a light white frost. After treatment, all patients were instructed to apply petrolatum ointment and sunscreen directly after the treatment and throughout the duration of treatment. Digital photographs were taken before and after each treatment. Time between treatments was variable and ranged from 1 to 10 months depending on each patient’s schedules. There was marked improvement in the size, number, and color of the lesions following treatment (Fig 1). No postinflammatory hyperpigmentation, hypopigmentation, significant swelling, or other adverse events were noted. Postprocedural monitoring ranged from 4 to 24 months during which time no recurrences were noted.

Case 1 is a 70-year-old male with numerous brown patches and plaques consistent with seborrheic keratoses on the face (Table I). He had previously used fluocinolone acetonide 0.01%, hydroquinone 4%, and tretinoin 0.05% combination cream without significant improvement. The patient underwent 5 treatments with a 532 nm picosecond laser over 2 years. The fluences used ranged between 0.7 and 0.8 J/cm^2^ with a 4 mm spot size.

**Table I:** 532-nm picosecond laser treatment settings for three patients

Case	Age	Gender	Race	Fitzpatrick skin type	Number of seborrheic keratoses	Number of laser treatments	Fluence (J/cm^2^)	Spot size (mm)	Range of total pulses	Overall treatment duration
1	70	M	Asian	IV	>150	5	0.7 – 0.8	4	230 to 344	2 y
2	67	M	Asian	IV	>75	3	1.2 – 1.4	3	86 to 141	5 mo
3	69	F	Asian	IV	>100	4	0.5 – 1.2	4	103 to 409	2 y

Case 2 is a 67-year-old male with seborrheic keratoses on the bilateral cheeks (Table I). He underwent 3 treatments with a 532 nm picosecond laser over the course of 5 months. The fluences used ranged between 1.2 and 1.4 J/cm^2^ using a 3 mm spot size.

Case 3 is a 69-year-old-female with seborrheic keratoses on the bilateral cheeks and temples. She had 4 treatments over the course of 2 years with a 532 nm picosecond laser (Table I). The fluences used ranged between 0.5 and 1.2 J/cm^2^ using a 4 mm spot size. The patient also used hydroquinone 6% cream concurrently.

## Discussion

These cases demonstrate the successful use of picosecond laser in the treatment of seborrheic keratosis in Asian skin types. The 532 nm wavelength was selected due to the superficial nature of these lesions and high melanin targeting. In contrast to the treatment of lentigines, high fluences can be tolerated because of the nature of seborrheic keratosis being lesions at the epidermal surface that absorb the energy with relative sparing of endogenous melanin.^6^ Ensuring effective counseling with patients about periprocedural and postprocedural care facilitates a more successful outcome while reducing the risk of adverse events.

Despite the known additional risk of postinflammatory hyperpigmentation and hypopigmentation, skin texture changes, or scarring in patients with more pigmented skin types, these cases demonstrate the successful use of an ultrashort pulse duration (250-450 picosecond range) picosecond laser for the treatment of seborrheic keratoses in patients with skin of color and should be considered as a potential treatment option. Future directions include evaluation of a larger sample size and inclusion of a wider range of Asian skin phenotypes to further evaluate the safe use of laser treatment for seborrheic keratoses in skin of color.

## Conflicts of interest

None disclosed.
